# The Medical Society of the County of Monroe

**Published:** 1891-07

**Authors:** 


The Medical Society of the County of Monroe held its seventy-first annual meeting in Rochester, May 27, 1891, under the presidency of John O. Roe, M. D. There were nearly one hundred physicians present, and scientific papers were read by Doctors E. H. Howard, Marion Craig, P. D. Carpenter, E. W. Mulligan, Charles Forbes, Anna H. Searing, W. M. Hawkins, J. L. Roseboom, H. S. Durand, R. M. Moore, L. A. Weigel, F. F. Dow, W. L. Conklin, J. W. Whitbeck, A. Dann, H. T. Williams, A. W. Henckell, George W. Goler, and S. L. Elsner.

Dr. Roe chose for the subject of his presidental address, The Climatic Therapeutics of the Respiratory Organs with Special Consideration of the Climate of Western New York, which was dealt with in a masterly manner, and from the standpoint of a specialist skilled in the treatment of diseases of the respiratory tract.

Drs. E. W. Mulligan and W. J. Herriman were elected as delegates to the Medical Society of the State of New York, and Drs.

C. W. Wilbor and H. S. Durand, of Rochester, and J. W. Craig, of Churchville, were elected delegates to the American Medical Association.

Drs. F. F. Dow, E. B. Angell, and H. S. Durand were appointed a committee to consider the feasibility of establishing a medical library in Rochester. This was a model county society meeting, and it would be well for Erie county to take a lesson therefrom.

